# Biocontrol potential of lipopeptides produced by the novel *Bacillus subtilis* strain Y17B against postharvest *Alternaria* fruit rot of cherry

**DOI:** 10.3389/fmicb.2023.1150217

**Published:** 2023-03-15

**Authors:** Tanvir Ahmad, Fuguo Xing, Chengrong Nie, Changyu Cao, Ying Xiao, Xi Yu, Anam Moosa, Yang Liu

**Affiliations:** ^1^School of Food Science and Engineering, Foshan University, National Technical Center (Foshan) for Quality Control of Famous and Special Agricultural Products (CAQS-GAP-KZZX043), Guangdong Key Laboratory of Food Intelligent Manufacturing, Foshan, Guangdong, China; ^2^Key Laboratory of Agro-Products Quality and Safety Control in Storage and Transport Process, Ministry of Agriculture and Rural Affairs/Institute of Food Science and Technology, Chinese Academy of Agricultural Sciences, Beijing, China; ^3^School of Life Sciences and Engineering, Foshan University, Foshan, Guangdong, China; ^4^Faculty of Medicine, Macau University of Science and Technology, Macao SAR, China; ^5^Department of Plant Pathology, The Islamia University of Bahawalpur, Bahawalpur, Pakistan

**Keywords:** postharvest disease, fruit rot, *Bacillus subtilis*, biocontrol, antifungal, *Alternaria alternata*

## Abstract

The use of synthetic fungicides against postharvest *Alternaria* rot adversely affects human health and the environment. In this study, as a safe alternative to fungicides, *Bacillus subtilis* strain Y17B isolated from soil exhibited significant antifungal activity against *Alternaria alternata.* Y17B was identified as *B. subtilis* based on phenotypic identification and 16S rRNA sequence analysis. To reveal the antimicrobial activity of this strain, a PCR-based study detected the presence of antifungal lipopeptide (LP) biosynthetic genes from genomic DNA. UPLC Q TOF mass spectrometry analysis detected the LPs surfactin (m/z 994.64, 1022.68, and 1026.62), iturin (m/z 1043.56), and fengycin (m/z 1491.85) in the extracted LP crude of *B. subtilis* Y17B. *In vitro* antagonistic study demonstrated the efficiency of LPs in inhibiting *A. alternata* growth. Microscopy (SEM and TEM) studies showed the alteration of the morphology of *A. alternata* in the interaction with LPs. *In vivo* test results revealed the efficiency of LPs in reducing the growth of the *A. alternata* pathogen. The overall results highlight the biocontrol potential of LPs produced by *B. subtilis* Y17B as an effective biological control agent against *A. alternata* fruit rot of cherry.

## 1. Introduction

*Alternaria alternata* is a common storage pathogen that causes considerable postharvest contamination and rot of fruit crops. It can grow rapidly at low temperatures and causes food spoilage during transport and storage (Ostry, 2008; Xu et al., 2018). *Alternaria* species are reported to produce more than 60 different secondary metabolites, including mutagenic *Alternaria* mycotoxins, during their growth on the substrate, which leads to a high risk of human and animal health hazards (Pruß et al., 2014; Andersen et al., 2015). Every year, a large amount of food and feedstuff is contaminated and spoiled due to the growth of *Alternaria* species and their toxins. Additionally, previous studies have reported that *A. alternata* is associated with asthma (Sanchez and Bush, 2001). Owing to the great damage caused by *Alternaria* species, it is necessary to study and develop an effective management strategy for this fungus. Currently, the use of fungicides is the most widely used method to control *A. alternata* (Soylu and Kose, 2015) but their application has a long-term residual effect, which poses a high potential risk to health, food safety, and environmental pollution (Özkara et al., 2016).

Biological control is regarded as a reliable and safe alternative to chemicals (Li et al., 2016). Some bacterial species have been reported as promising biological control agents due to their strong antagonistic activity. They possess multiple modes of action and produce a wide variety of biologically active compounds with antifungal potential against different preharvest and postharvest diseases (Gond et al., 2015; Mnif et al., 2016). Among these antagonistic bacteria, *Bacillus* is well known for its production of broad-spectrum antimicrobial compounds, plant growth promotion, enhancement of plant biomass, and induction of systemic resistance in plants against plant pathogenic fungi (Ongena and Jacques, 2008; Alina et al., 2015; Fatima et al., 2023). *Bacillus* species have recently emerged as an interesting source of biocontrol agents for postharvest fungal disease management. These species showed different antifungal mechanisms, including the production of antifungal volatile organic compounds (VOCs) and lipopeptides (LPs), the induction of disease resistance, and nutrient competition (Wang et al., 2022b). *B. amyloliquefaciens*, *B. subtilis*, *B. licheniformis*, and *B. cereus* have been reported to produce LPs with a high antifungal activity (Lawrance et al., 2014; Zhao et al., 2017; Basit et al., 2018; de Souza et al., 2018). Three families of LPs, iturin, surfactin, and fengycin, have been reported to be inhibitory against pathogenic fungi and oomycetes (Stein, 2005; Ongena and Jacques, 2008). Based on their structures, iturin is a cyclopeptide consisting of C14 to C17, β-amino fatty acids, and heptapeptides. Surfactin comprises cyclic lactone rings (C13 to C16), β-hydroxy fatty acids, and heptapeptides. Fengycin is a decapeptide and is composed of β-hydroxy fatty acid chains, which form cyclic lactone rings (Ahmad et al., 2020). The antimicrobial potential of LPs is closely related to the peptide cyclization, amino acid residual sequence, and branching length of the fatty acid chain (Zhao et al., 2018). LPs are low molecular weight compounds that are synthesized by a complex of multi-enzymes, called non-ribosomal peptide synthetases (NRPSs). Most of the short sequence peptides, generally less than 50 amino acids, have been mostly identified as antimicrobial peptides. Therefore, NRPSs can generate a range of variants based on their amino acid sequence and fatty acid chain length. *Bacillus* species can simultaneously produce different families of LPs and their isoforms, i.e., surfactin, fengycin, and iturin (Finking and Marahiel, 2004; Montesinos, 2007; Marahiel, 2009; Han et al., 2015; Yang et al., 2015; Deng et al., 2017).

The antifungal potential of LPs has been reported against several postharvest fungal pathogens (Arrebola et al., 2010; Arroyave-Toro et al., 2017; Cozzolino et al., 2020). However, the antifungal potential of LPs produced by *B. subtilis* against *A. alternata* has not yet been systematically studied. To understand the mechanism of LPs against fruit decay of postharvest cherry caused by *Alternaria*, we were prompted to evaluate its antifungal potential. The primary objective of the present study was (a) to isolate a *Bacillus* strain with promising antifungal potential against *A. alternata*, (b) to identify genes in the *B. subtilis* Y17B genome that are involved in synthesizing antifungal LPs, (c) to identify antifungal LPs in the crude extract of Y17B on the basis of molecular weight using ultra-high-performance liquid chromatography-quadrupole time-of-flight mass spectrometry (UPLC Q TOF-MS) and test their combined antifungal activity *in vitro*, and (d) to examine alterations of the morphology of *A. alternata* upon interaction with these LPs using scanning electron microscopy (SEM) and transmission electron microscopy (TEM). Furthermore, the biocontrol potential of LPs was tested on cherry fruit to demonstrate the ability of LPs to protect it against *A. alternata* infection. To the best of our knowledge, this is the first report to explain the efficiency of LP activity in enhancing the shelf life of cherry fruit against *A. alternata*.

## 2. Materials and methods

### 2.1. Isolation of bacterial strains and fungal culture

The soil samples were collected from Tengchong (Thermal Sea area), Baoshan City, Yunnan Province, China in 2019. Isolation was carried out using a method described previously (Chen et al., 2017) with slight modification. Each soil sample (5 g) was placed in a 100-ml flask containing 50 ml of sterile water mixed with 0.1% NaCl to make the sample suspensions a concentration of 10^−1^, which were then incubated overnight at room temperature. The sample suspensions were adjusted to concentrations of 10^−4^ and 10^−6^ through serial dilution, and 10 μl of each concentration was streaked on Luria–Bertani (LB) agar medium and incubated at 37°C for 4 days. Single purified colonies were obtained by re-streaking two times and preserved in 25% glycerol at −18°C. The single colonies were further screened for their ability to produce antifungal agents. *Alternaria alternata* ACT-1, isolated by Ahmad et al. (2020) and associated with postharvest fruit rot of cherry, was preserved in 25% glycerol solution and occasionally retrieved and grown on potato dextrose agar (PDA) medium prior to conducting experiments.

### 2.2. Antifungal assay

The antifungal effect of bacterial strains was evaluated in a dual culture assay described previously (Wu et al., 2019) with slight modification. A mycelium plug (5 mm) was taken from a 7-day-old culture of *A. alternata* and placed at the center of the PDA plates. Then, 10 μl of bacterial culture was inoculated 30 mm away from the mycelium plug and incubated at 28°C for 7 days post inoculation (dpi). To assess antifungal activity, the percentage of growth inhibition was calculated using the following formula described by Vincent (1947) at 7 dpi:

I = [(C−T)/C] × 100

where I is the percentage of growth inhibition, C is growth in control, and T is growth in treatment.

### 2.3. Morphological, biochemical, and physiological characteristics of *Bacillus subtilis* Y17B

For morphological characterization, *B. subtilis* Y17B culture (10 μl) was streaked on an LB agar plate and incubated at 37°C for 4 days. The colony patterns were observed under a stereomicroscope. For SEM study, the specimen was washed twice with phosphate buffer, then fixed with 2.5% glutaraldehyde buffer and stored at 4°C for 24 h. Then, the specimen was rinsed three times with 100 mM phosphate buffer sequentially for 5 min and postfixed in 1% osmium tetroxide fixation solution for 2 h. Next, the specimen was subjected to gradient dehydration with 30, 50, 60, 70, 80, 90, 95, and 100% ethanol solution sequentially for 15 min. The specimen was dried with a Leica EM CPD030 automated dryer (Leica Microsystems Inc., Germany), attached to a sample stage, and sputtered with gold coating by ion sputtering (HITACHI MC1000). Morphology was observed using a HITACHI-SU8010 SEM (Hitachi High-Technologies Corporation, Tokyo, Japan). Biochemical and physiological identification of strain Y17B was carried out following the instructions featured in Bergey’s manual of systematic bacteriology (Vos et al., 2011).

### 2.4. Molecular identification and phylogenetic analysis

**Figure 1:**
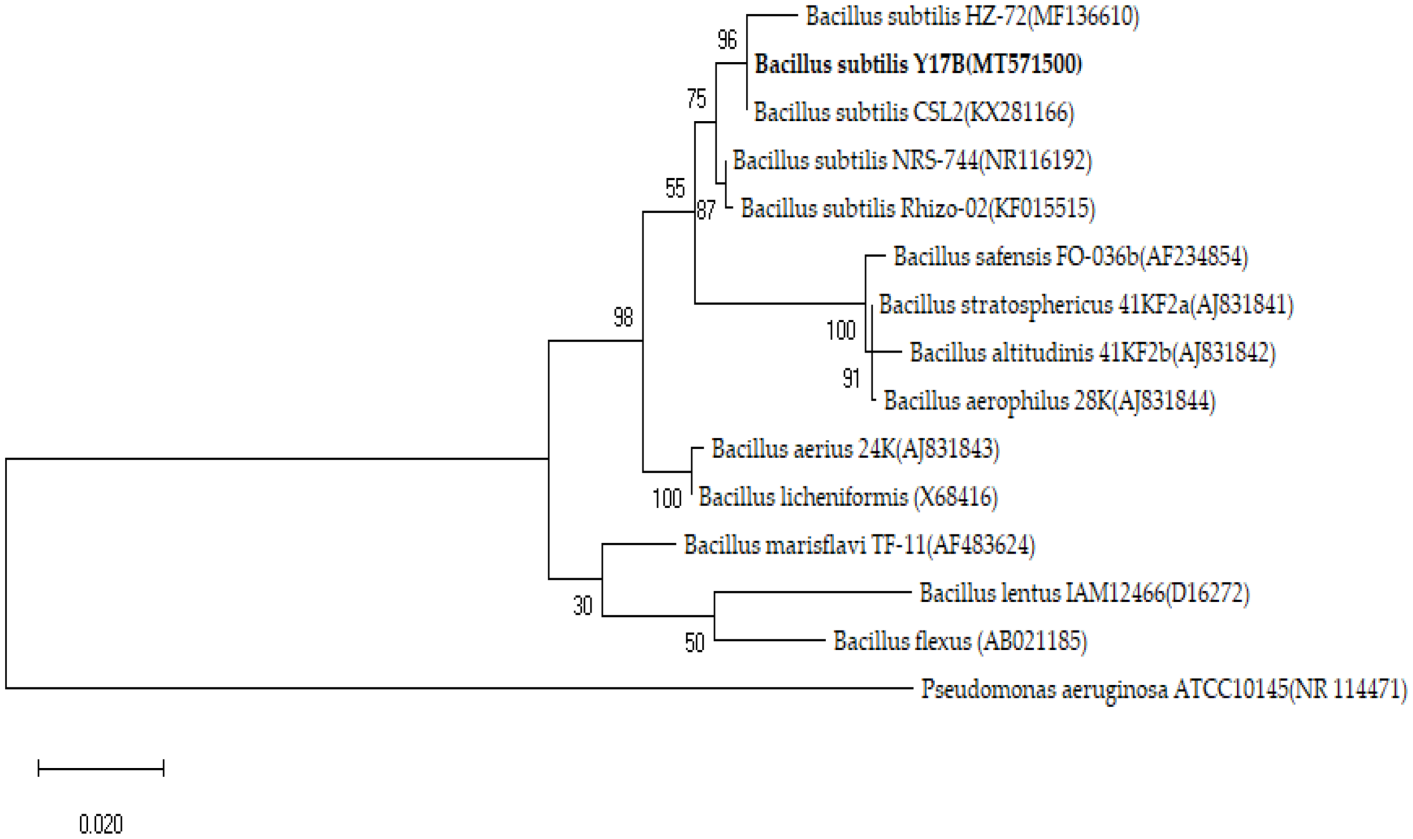
Maximum likelihood phylogenetic tree of the tested bacteria based on the Taimura Nei model. The scale bar represents 0.02 nucleotide substitutions per nucleotide position. The numbers on the nodes are bootstrap values inferred from 1,000 replicates. The reference isolates used in this study were as follows: *B. aerophilus* 28 K (GenBank ID. AJ831844), *B. altitudinis* 41KF2b (AJ831842), *B. stratosphericus* 41KF2a (AJ831841), *B. licheniformis* (X68416), *B. aerius* 24 K (AJ831843), *B. subtilis* HZ-72 (MF136610), *B. safensis* FO-036b (AF234854), *B. subtilis* Rhizo-02 (KF015515), *B. subtilis* CSL2 (KX281166), *B. flexus* (AB021185), *B. marisflavi* TF-11 (AF483624), *B. lentus* IAM12466 (D16272), *B. subtilis* NRS-744 (NR116192), *B. licheniformis* (X68416), and *Pseudomonas aeruginosa* ATCC10145 (NR_114471).

Genomic DNA of *B. subtilis* Y17B was extracted using a bacterial DNA kit D3350-01 (Omega Bio-Tek) according to the manufacturer’s protocol. The concentration and purity of the DNA was measured using a NanoDrop ND-1000 spectrophotometer (Thermo Fisher Scientific, Massachusetts, United States). The 16S rRNA gene was amplified using the primers 27F (AGAGTTTGATCCTGGCTCAG) and 1492R (CTACGGCTACCTTGTTACGA; Hou et al., 2018). The PCR (BIO-RAD T100™) conditions were as follows; initial denaturation at 94°C for 3 min, followed by 34 cycles of denaturation at 94°C for 30 s, annealing at 58°C for 30 s, and extension at 72°C for 30 s/kb, and a final extension at 72°C for 5 min. The PCR product was sequenced by Personal bio (Shanghai Personal Biotechnology Co., Ltd., China). The obtained sequences were subjected to an NCBI BLAST similarity search. For phylogenetic analysis, the published reference sequences already available in the NCBI database that were most homologous to the DNA sequences of *B. subtilis* Y17B strain were used for alignments. GenBank accession numbers and the complete record of isolates from NCBI are listed in the caption of Figure 1. The concatenated sequences and the outgroup were analyzed using Molecular Evolutionary Genetics Analysis (MEGA Version X; Kumar et al., 2018). The sequence alignment of genes was performed using Clustal W. The evolutionary history was inferred by constructing a maximum likelihood tree (ML; Saitou and Nei, 1987) of 16S rRNA gene sequences based on the Tamura Nei model (Tamura and Nei, 1993). The confidence values for clade stability within the phylogenetic tree were determined using bootstrap analysis with 1,000 replicates of the aligned sequences.

### 2.5. PCR amplification of lipopeptide biosynthetic genes from genomic DNA

Genomic DNA extraction was carried out using a bacterial DNA kit D3350-01 (Omega Bio-Tek) according to the manufacturer’s protocol. DNA amplification was carried out using specifically designed primers for LP biosynthetic genes: surfactin, fengycin, and iturin (Table 1). Primer specificity was checked using the NCBI nucleotide BLAST tool. PCR amplification of genomic DNA was carried using a TRAN kit (Trans-Gen Biotech, China) as per the manufacturer’s protocol. The PCR conditions were as follows; initial denaturation at 94°C for 3 min, followed by 34 cycles of denaturation at 94°C for 30 s, annealing at 50°C for 30 s, and extension at 72°C for 30 s/kb, and a final extension at 72°C for 5 min. A negative control was also included in the PCR amplification reaction. The amplified PCR product from each reaction was visualized on a 1% agarose gel stained with ethidium bromide.

**Table 1:** Primers used in this study to amplify the lipopeptide biosynthetic genes through PCR.

Sr.#	Lipopeptides	Melting temperature (°C)	Fragment size (bps)	Sequence	References
1	Surfactin	56.3	1,298	CGGTGATCTTGCGAAGCTTTAT CGCTTTCGTTCTGCCATTCT	Farzand et al. (2019)
2	Iturin	55.9	1,244	ACCTCACCTTGATCGGCTATAC TGGTGGGCGAAAGAAGTTTATG	Farzand et al. (2019)
3	Fengycin	56.3	1,447	CGGCCATTCGCTCATCTTTAT GTTTCCGCTTCATCAGTCTCTTC	Farzand et al. (2019)

### 2.6. Extraction of crude lipopeptides from *Bacillus subtilis* Y17B

The crude extract of LPs were extracted using a method described previously (Gu et al., 2017) with slight modification. *B. subtilis* Y17B was inoculated into 50 ml of LB liquid medium and incubated for 24 h at 37°C with shaking at 180 rpm. Y17B culture (50 ml) was taken and inoculated into 3 l of Landy medium and incubated at 37°C with shaking at 180 rpm for 2 days. The supernatant was collected by centrifugation (Eppendorf centrifuge 5804R, Germany) at 12,000 × g for 20 min at 4°C. LPs in the supernatant were precipitated by adjusting the pH to 2 and kept for 6 h at room temperature. The precipitates were collected by centrifugation at 12,000 × g for 20 min at 4°C and dissolved in HPLC grade methanol (Fisher Chemicals, United Kingdom). The pH of the solution was adjusted to 7 by adding 1 M NaOH and the mixture was vortexed for 1 min followed by centrifugation at 4,000 × *g* for 5 min. The supernatant was passed through a silica gel column and eluted with a mixed solution of HPC grade methanol and methylene chloride at a 5:1 ratio, respectively, as a mobile phase with a 1 ml/min flow rate. The eluted solution was dried under a gentle stream of pure nitrogen at 45°C and the LPs were harvested.

### 2.7. Identification of antifungal lipopeptides by ultra-high-performance liquid chromatography-quadrupole time-of-flight mass spectrometry

The extracted LPs were dissolved in HPLC grade acetonitrile (ACN) to obtain 50 μg/ml concentrated crude LP extract solution, which was analyzed by UPLC Q TOF-MS to identify the antifungal agents and their isomers. UPLC Q TOF-MS was performed using a Waters ACQUITY UPLC® system (Waters Co., United States) equipped with a binary solvent manager FTN delivery system, auto-sampler manager, and detector. Chromatographical separation was achieved using an ACQUITY UPLC® HSS T3 1.8 μm (2.1 × 100 mm) Column (Part No. 186003539; Serial No. 02163904225158). The mobile phase contained (A) 0.1% aqueous formic acid (v/v) and (B) HPLC grade acetonitrile (ACN). The gradient elution conditions of UPLC were adjusted as follows; 0–7% B (0–11 min), 7–12% B (11–13.9 min), 12–15% B (13.9–14 min), 15–34% B (14–29 min), 34–70% B (29–32 min), 70–100% B (32–35 min), 100% B (35–42 min), 100–0% B (42–45 min), and 0% B (45–60 min). The flow rate was 0.3 ml/min. The pressure range was set at 0 psi (minimum) to 13,000 psi (maximum). The temperature of the column and auto-sampler was maintained at 35°C and 10°C, respectively. An aliquot of 5 μl of the sample was injected and mass spectrometry (MS) analysis was performed using a Waters Xevo-G2QTOF-MS. The MS (Waters Co., United States) was equipped with an electrospray ionization (ESI) source operating in positive ionization mode. The collision energy and ramp collision energy were set at 6 V and 20 to 30 V, respectively. The capillary voltage and cone voltage were set at 2.23 kV and 40 V, respectively. The source and desolvation temperatures were set at 100°C and 500°C, respectively. The desolvation gas flow was set at 995 l/h at a temperature of 450°C. The cone gas was set at 50 l/h. The Q_TOF acquisition rate was 0.2 s and the inter-scan delay was 0.1 s. A mass range was scanned from 200 to 2000 Da. All data were collected using Mass-Lynx V4.1. software (Waters Co., United States).

### 2.8. Antifungal activity assay of lipopeptides using a dual culture technique

*A. alternata* mycelial plugs (5 mm) were taken from 7-day-old culture and placed in the center of fresh PDA plates. Subsequently, 10 μl of 300 μg/ml LP solution was prepared with 0.01 M PBS buffer and inoculated 30 mm away from the fungal mycelial block on a sterilized filter paper block. For the control treatment, only 10 μl of PBS was inoculated 30 mm away from the fungal block. The Petri plates were sealed and incubated at 28°C for 7 days. The percentage of growth inhibition was measured using the following formula described by Vincent (1947) at 7 dpi:

I = [(C−T)/C] × 100

where I is the percentage of growth inhibition, C is growth in control, and T is growth in treatment.

#### 2.8.1. Anti-mycelial assay in potato dextrose agar amended with lipopeptides

PDA was autoclaved and precooled to 40–45°C and poured onto 90 mm plates. LP solution (1.5 ml) at different concentrations (50, 100, 200, 300, and 500 μg/ml) was mixed into the PDA plates. The control treatment contained 1.5 ml of PBS only. After solidification of the PDA, a mycelial plug (5 mm) from a 7-day-old culture of *A. alternata* was placed in the middle of each Petri plate and incubated at 28°C for 7 days. The percentage of growth inhibition was calculated using the following formula described by Vincent (1947) at 7 dpi:

I = [(C−T)/C] × 100

where I is the percentage of growth inhibition, C is growth in control, and T is growth in treatment. The experiment was repeated three times.

### 2.9. Ultrastructure analysis of *Alternaria alternata*

The ultrastructural changes in the pathogen were studied using a protocol described previously (Wu et al., 2019) with modification. An aliquot of 10 μl of 300 μg/ml LPs was inoculated on sterilized filter paper (5 mm) and placed 30 mm away from the fungal mycelial block and incubated for 7 dpi. Fungal mycelium was taken from the edge of the inhibition zone produced by the LPs. For the control treatment, sample was taken from the edge of mycelial growth, which was inoculated with only 10 μl of PBS. Samples were fixed into 2.5% glutaraldehyde and stored at 4°C for 48 h. For SEM analysis, the samples were prepared using the same procedure as described above in the ‘Morphological, Biochemical, and Physiological Characteristics of *B. subtilis* Y17B’ section. The samples were observed using a HITACHI SU8010 SEM (Hitachi High-Technologies Corporation, Tokyo, Japan). For TEM analysis, the samples were embedded in Epon 812 and then kept in an oven for 7 days for polymerization. The ultrathin sections were collected and analyzed using a HITACHI-7500 Bio-Transmission electron microscope (Hitachi High-Technologies Corporation, Tokyo, Japan).

### 2.10. Antagonism assay in cherry fruit

To evaluate the antifungal effect of LPs against *A. alternata* infection on cherry fruit, an antagonism assay was conducted. For this experiment, medium-sized healthy cherry fruit (Lapins cultivar) were selected and dipped in 1% NaOCl for 60 s and later rinsed three times with sterilized water for surface disinfection. Fruits were punctured with a sterilized needle to approximately make 3 mm × 2 mm (depth × width) wounds. Each wound was inoculated with a 10-μl suspension of *A. alternata* (10^8^ conidia/ml). Subsequently, 10 μl of 300 μg/ml LP solution was applied to the wounds. Inoculated fruits with only a 10-μl suspension of *A. alternata* were used as a disease control group. All fruits were placed in sterilized six-well culture cluster flat-bottom plates (Costar 3,516) and covered with a zipper bag (240 mm × 170 mm). The samples were incubated for 7 dpi at 28°C. Disease progress was evaluated by measuring the size (mm) of the lesions on the surface of the cherry fruit 7 dpi. The experiment consisted of a cherry fruit with three replicates per treatment.

### 2.11. Statistical analysis

The experiments were performed in a completely randomized design and subjected to one-way ANOVA, and the means were separated by Fisher’s least significant difference (LSD) test (*p* ≤ 0.05) using the statistical package Statistix (Ver. 8.1).

## 3. Results

### 3.1. Isolation and screening of *Bacillus subtilis* Y17B

**Figure 2:**
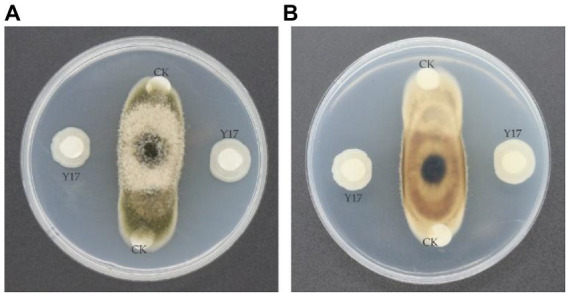
Antifungal activity of the Y17B strain against *A. alternata*. **(A,B)** Front **(A)** and reverse **(B)** side of the dual culture plate 7 dpi. CK, control (only LB medium); Y17, inoculated with *Bacillus subtilis* Y17B.

A total of 63 isolated bacterial strains from soil samples were evaluated for their ability to inhibit the fungal growth of *A. alternata* in a dual culture assay. Among all the strains, *B. subtilis* Y17B significantly inhibited the mycelial growth of *A. alternata.* Subsequently, 10 μl (cfu 10^7^/ml) of fresh *B. subtilis* Y17B culture inhibited fungal mycelial growth by 61.3% at 7 dpi (Figure 2).

### 3.2. Morphological and biochemical characteristics of *Bacillus subtilis* Y17B

**Figure 3:**
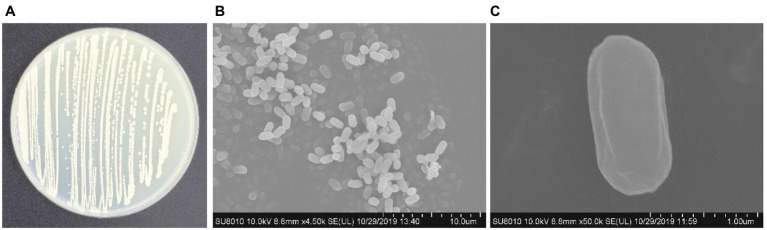
Morphological features of *B.* subtilis Y17B. **(A)** Colony morphology. **(B)** Bacterial cells in chain form. **(C)** Single bacterium. Scale bars: 10 μm **(B)**; 1 μm **(C)**.

The colony of *B. subtilis* Y17B was off white to creamy with irregular margins, sticky and had a smooth surface on the LB agar. Under a stereomicroscope, the colonies were joined together and appeared as a chain. SEM revealed that Y17B cells were straight and rod-shaped with round ends, organized in chains, and motile (Figure 3). The bacterial cell was 1.47 to 1.76 μm in length and 765 to 920 nm in width. The results of the biochemical identification are presented in Table 2. *B. subtilis* Y17B showed a positive result in catalase, citrate, Voges–Proskauer (VP), and nitrate reduction tests, and a negative result in a Methyl red (MR) test. Additionally, *B. subtilis* Y17B was able to utilize carbon substrates, including D-glucose, D-xylose, and sucrose.

**Table 2:** Biochemical and physiological tests of Y17B.

Test	Result
Catalase test	+
Citrate test	+
Voges–Proskauer (VP) test	+
Methyl red (MR) test	−
Nitrate reduction test	+
D-xylose	+
D-glucose	+
Sucrose	+

**+**, sign indicates positive; −, sign indicates negative.

### 3.3. Molecular identification and phylogenetic analysis of *Bacillus subtilis* Y17B

Based on the 16S rRNA gene, the Y17B strain was identified as *B. subtilis* with >99.9% similarity with *B. subtilis* strain CSL2 (GenBank ID, KX281166). Phylogenetic analysis of concatenated sequences of the 16S rRNA gene resulted in an ML tree consisting of four distinct clades. Y17B was grouped with *B. subtilis* strains in one clade along with other isolates of *Bacillus* species. *Pseudomonas aeruginosa* ATCC10145 was used as an outgroup and formed a separate clade (Figure 1). Therefore, based on molecular and phylogenetic analysis, the tested bacterium was described as *Bacillus subtilis* strain Y17B (GenBank ID, MT571500).

### 3.4. PCR amplification of antifungal lipopeptide biosynthetic genes from the genomic DNA of *Bacillus subtilis* Y17B

**Figure 4:**
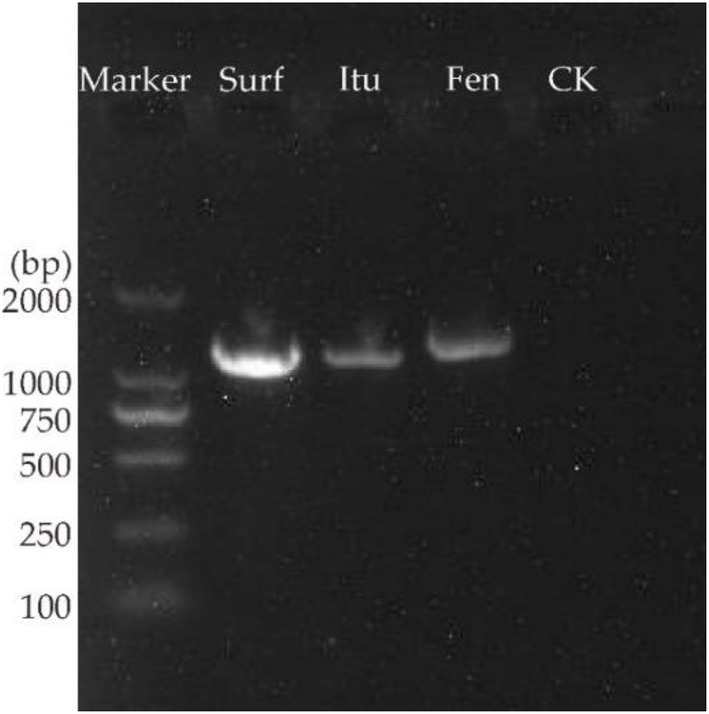
PCR amplification of antifungal LPs biosynthetic genes in *B. subtilis* Y17B. Surf, surfactin; Itu, iturin; Fen, fengycin; CK, control (without template DNA).

PCR amplification of surfactin, iturin, and fengycin from the genome of *B. subtilis* Y17B indicated the presence of LP biosynthetic genes. The gel showed the bands of all antifungal LPs according to their primer fragment size (bps), in contrast to the negative control without template DNA, which did not produce any band (Figure 4). The amplified products of the surfactin (band size of approximately 1,298 bps), iturin (approximately 1,244 bps), and fengycin (approximately 1,447 bps) genes were detected.

### 3.5. Identification of antifungal lipopeptides through ultra-high-performance liquid chromatography-quadrupole time-of-flight mass spectrometry analysis

**Figure 5:**
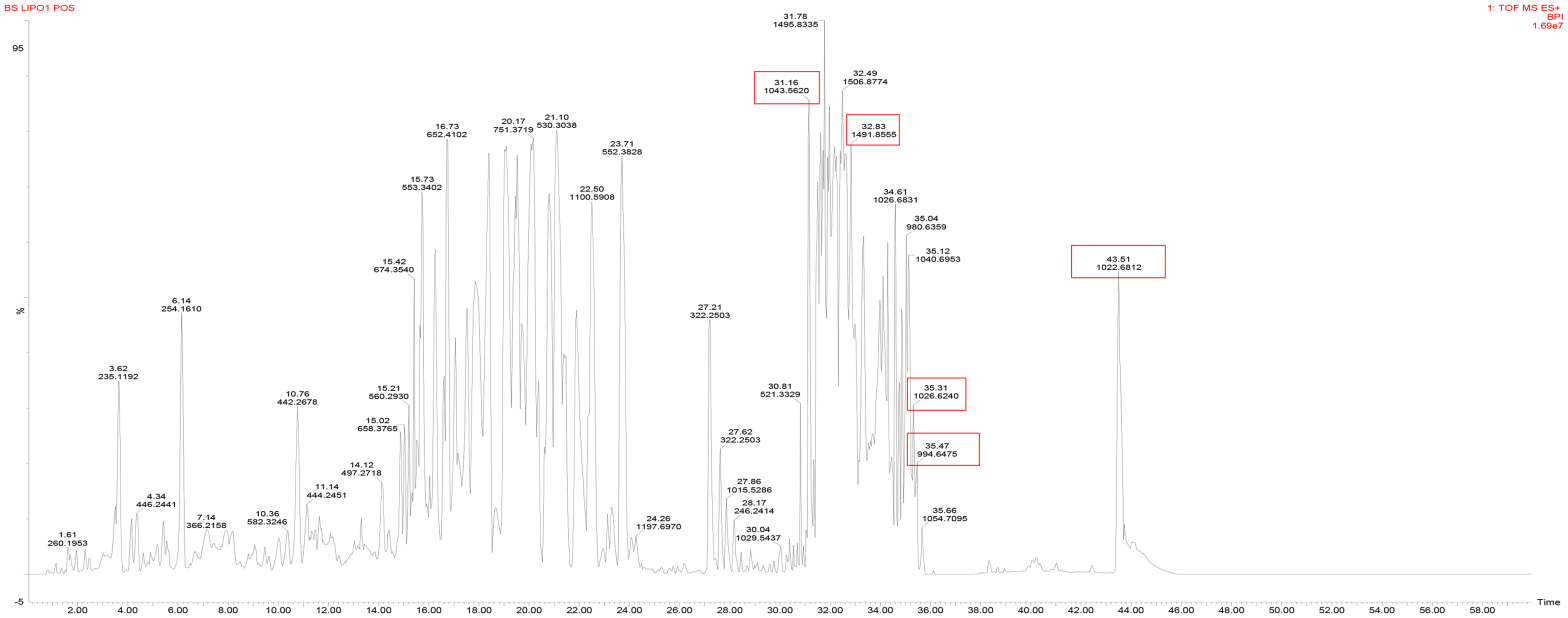
Total ion chromatogram of Y17B LPs with their molecular weight (m/z) and retention time (RT).

UPLC Q TOF-MS analysis was performed to identify the metabolites produced by *B. subtilis* Y17B. Figure 5 presents the total ion chromatogram (TIC) of LPs detected from crude extract of *B. subtilis* Y17B, which confirms the presence of three major and dominant antifungal LP families, surfactin, iturin, and fengycin, and their homologs. Three known surfactins with an acyl fatty chain length ranging from C12 to C14 were detected, along with one known iturin (C14). There were two peaks corresponding to fengycin (C16), and bacillomycin was not observed. Two homologs of surfactin [M + H] ^+^ were detected at 994.64 and 1022.681 m/z (retention time, 35.47 and 43.51 min, respectively). Iturin was identified [M + H] ^+^ at 1043.56 m/z (retention time, 31.16 min) and fengycin was observed [M + H] ^+^ at 1491.84 m/z (retention time, 32.12 min). LPs were identified by comparing their masses with those previously reported in the literature for respective compounds (Table 3).

**Table 3:** UPLC-QTOF-MS analysis of LPs produced by Y17B.

Lipopeptides	Fatty chain length	Mode	Current experimental mass	Previously reported mass	References
Surfactin	C12	[M + H] ^+^	994.64	994.64	Toral et al. (2018)
Surfactin	C14	[M + H] ^+^	1022.68	1022.67	Toral et al. (2018)
Surfactin	C14	[M + Na] ^+^	1026.62	1026.0	Sarwar et al. (2018)
Iturin	C14	[M + H] ^+^	1043.56	1043.50	Pathak and Keharia (2014); Zhao et al. (2018)
Fengycin	C16	[M + H] ^+^	1491.85	1491.84	Toral et al. (2018)

### 3.6. Dual culture assay

**Figure 6:**
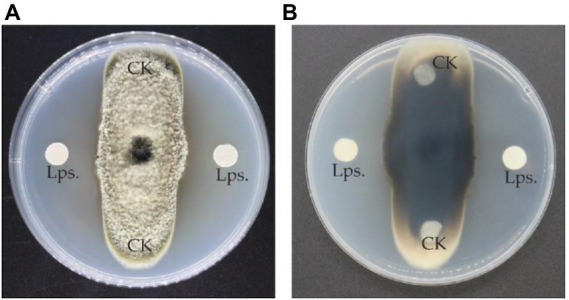
Antagonistic interaction of LPs with the pathogen. **(A,B)** Front **(A)** and reverse side **(B)** of the dual culture plate.

The antifungal LPs significantly inhibited *A. alternata* growth. The fungal growth of *A. alternata* was reduced by 54% in the dual culture assay by treatment with 10 μl of 300 μg/ml of LPs of *B. subtilis* Y17B. The growth of *A. alternata* was gradually reduced as the concentration of LPs of *B. subtilis* Y17B was increased (Figure 6).

#### 3.6.1. Antifungal assay on potato dextrose agar amended with lipopeptides

**Figure 7:**
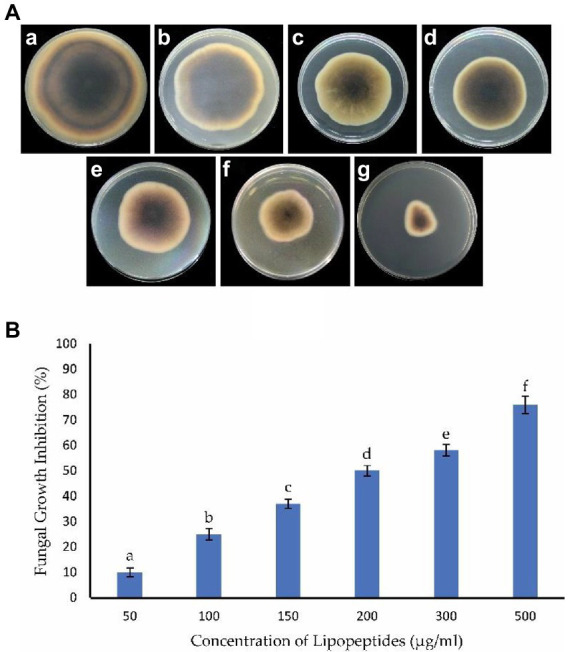
Antagonistic effect of *B. subtilis* Y17B LPs against *A. alternata*. **(A)** Assessment of LPs at different concentrations against *A. alternata*. The reverse side of the plate is shown. **(A)** Control, **(B)** 50 μg/mL of LPs, **(C)** 100 μg/mL of LPs, **(D)** 150 μg/mL of LPs, **(E)** 200 μg/mL of LPs, **(F)** 300 μg/mL of LPs, and **(G)** 500 μg/mL of LPs. **(B)** Graph representing the antagonistic effect of LPs on *A. alternata* mycelium inhibition (%). The same letters are not significantly different from each other. Analyzed using a LSD test (*p* ≤ 0.05).

Antifungal activity was evaluated by amending the PDA medium with the crude extract of LPs. The LPs produced by *B. subtilis* Y17B significantly inhibited the mycelial growth of *A. alternata*. Fungal mycelial growth was inhibited 76, 58, 52, 37, 25, and 10% at 500, 300, 200, 150, 100, and 50 μg/ml concentrations of LPs, respectively, 7 dpi (Figure 7). Therefore, the crude extract of LPs from *B. subtilis* Y17B inhibited *A. alternata* in a dose-dependent manner.

### 3.7. Ultrastructural changes in the morphology of *Alternaria alternata* hyphae and conidia caused by antifungal lipopeptides

**Figure 8:**
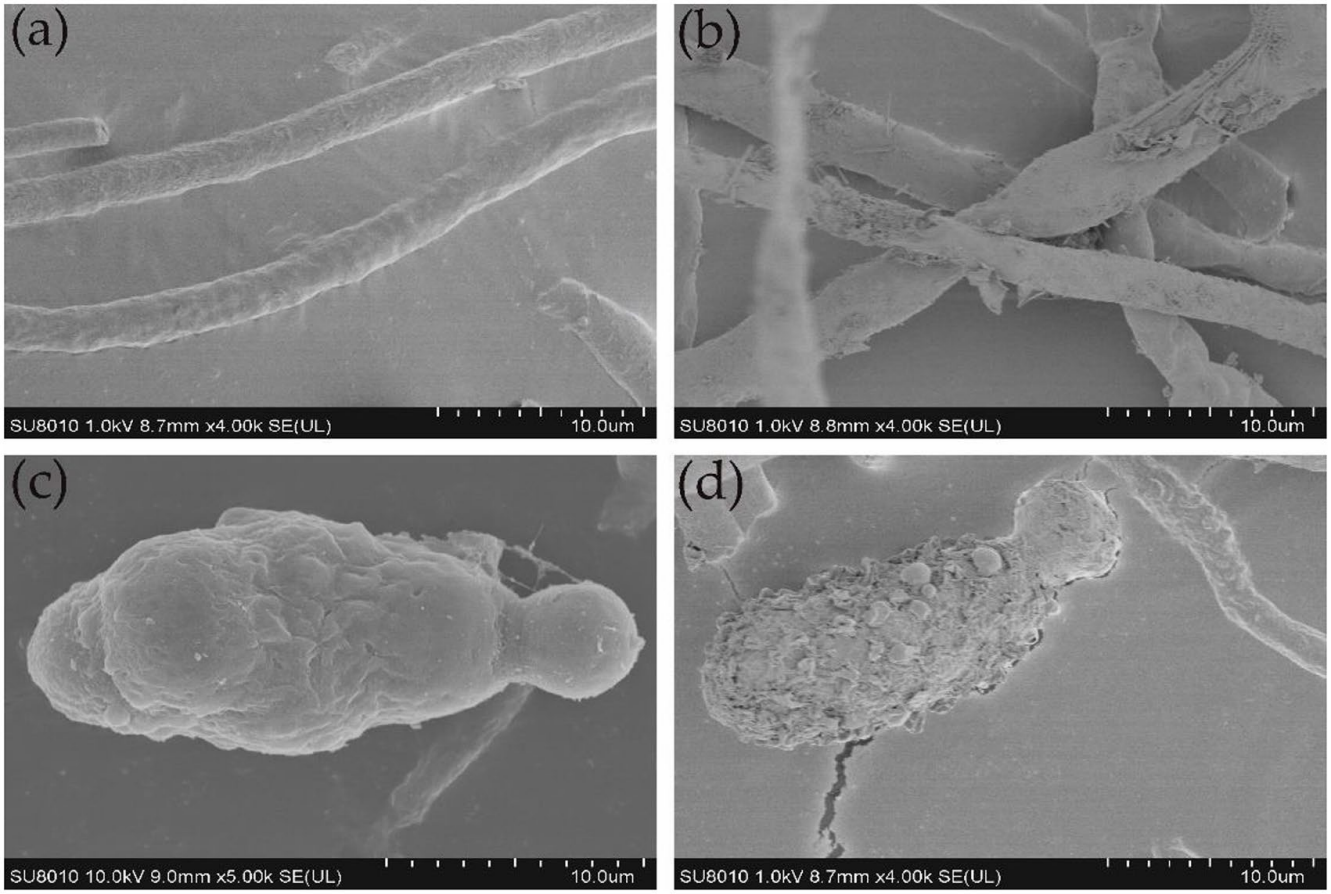
Ultrastructural changes in the hyphae and conidia of *A. alternata*. **(A)** Control group; healthy hyphae. **(B)** Effect of 10 μL of 300 μg/mL of LPs on hyphae. **(C)** Control group; healthy conidia. **(D)** Effect of 10 μL of 300 μg/ml of LPs on conidia.

**Figure 9:**
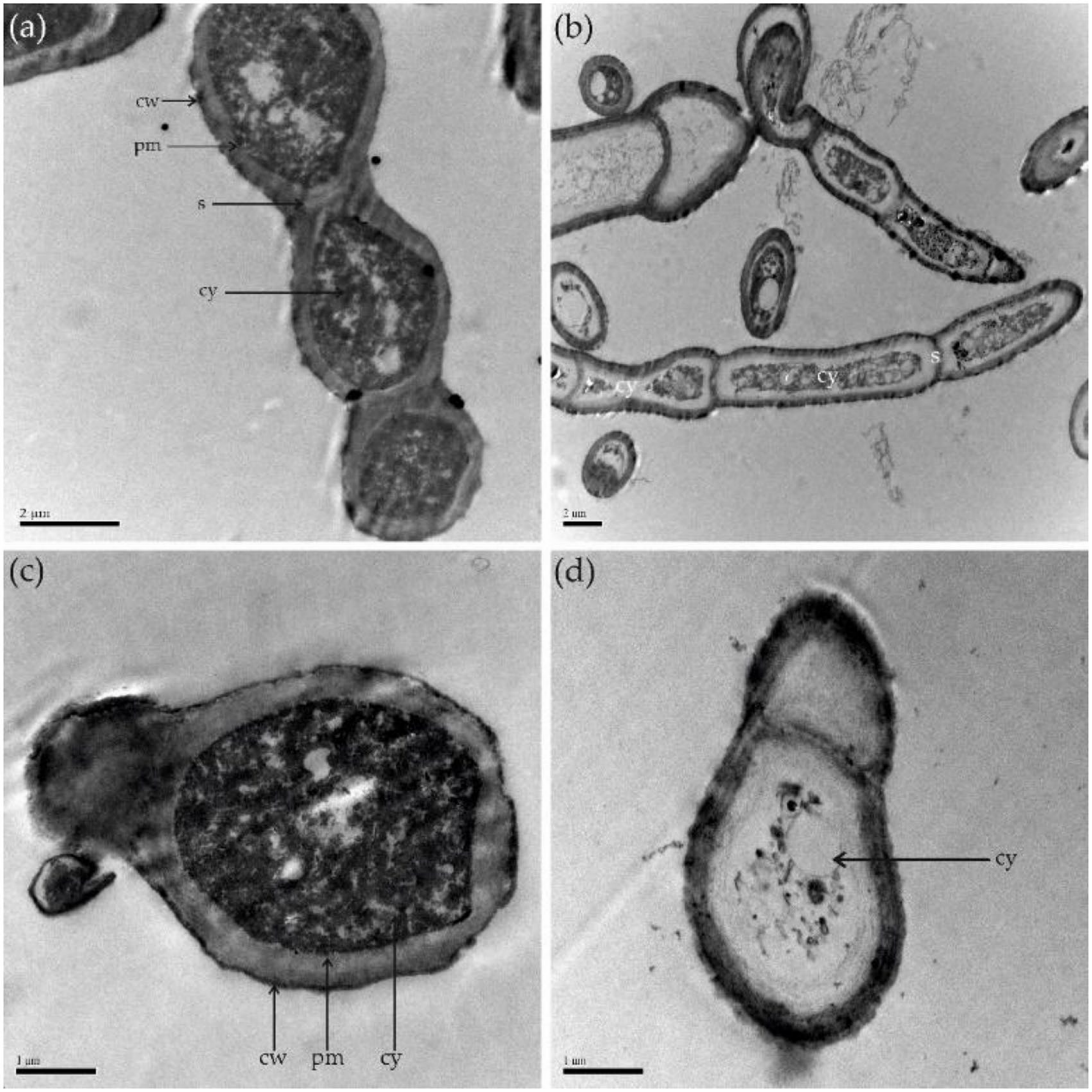
Transmission electron microscopy. **(A)** Control group; healthy hyphae. **(B)** Hyphae treated with 10 μl of 300 μg/ml of LPs. **(C)** Healthy conidia. **(D)** Conidia treated with 10 μL of 300 μg/mL of LPs. cw, cell wall; cy, cytoplasm; pm, plasma membrane; s, septum.

SEM analysis revealed that the untreated control hyphae of *A. alternata* seemed intact, regular, long, dense, and plump with a column-like trunk, and conidia were smooth, normal, and flask-shaped. However, the LP-treated hyphae and conidia showed alterations in structure, exhibiting irregular shapes, loose cell walls, ruptured cell wall surfaces, surface subsidence, and shriveled trunks. The results demonstrate the leakage of cellular contents due to LP treatment (Figure 8). TEM analysis was performed to observe internal structural alterations of the LP-treated pathogen. The untreated control hyphae and conidia showed distinct cell walls, intact plasma membranes, smooth surfaces, septa, and uniformly distributed and clearly visible cytoplasm. By contrast, the cell walls of the treated hyphae and conidia were thinner, representing the cytoplasm leakage. Cell walls showed irregular structure, such as a lack of discernible layers, uneven thickness, and gapped structures. Additionally, LP treatment caused plasmolysis and fragmentation of the plasma membranes. The cell membranes were dissolved and ruptured. Furthermore, the damage to the cell walls and membranes finally resulted in serious leakage of the cytoplasm, resulting in cell death (Figure 9).

### 3.8. Antagonism assay in cherry fruit

**Figure 10:**
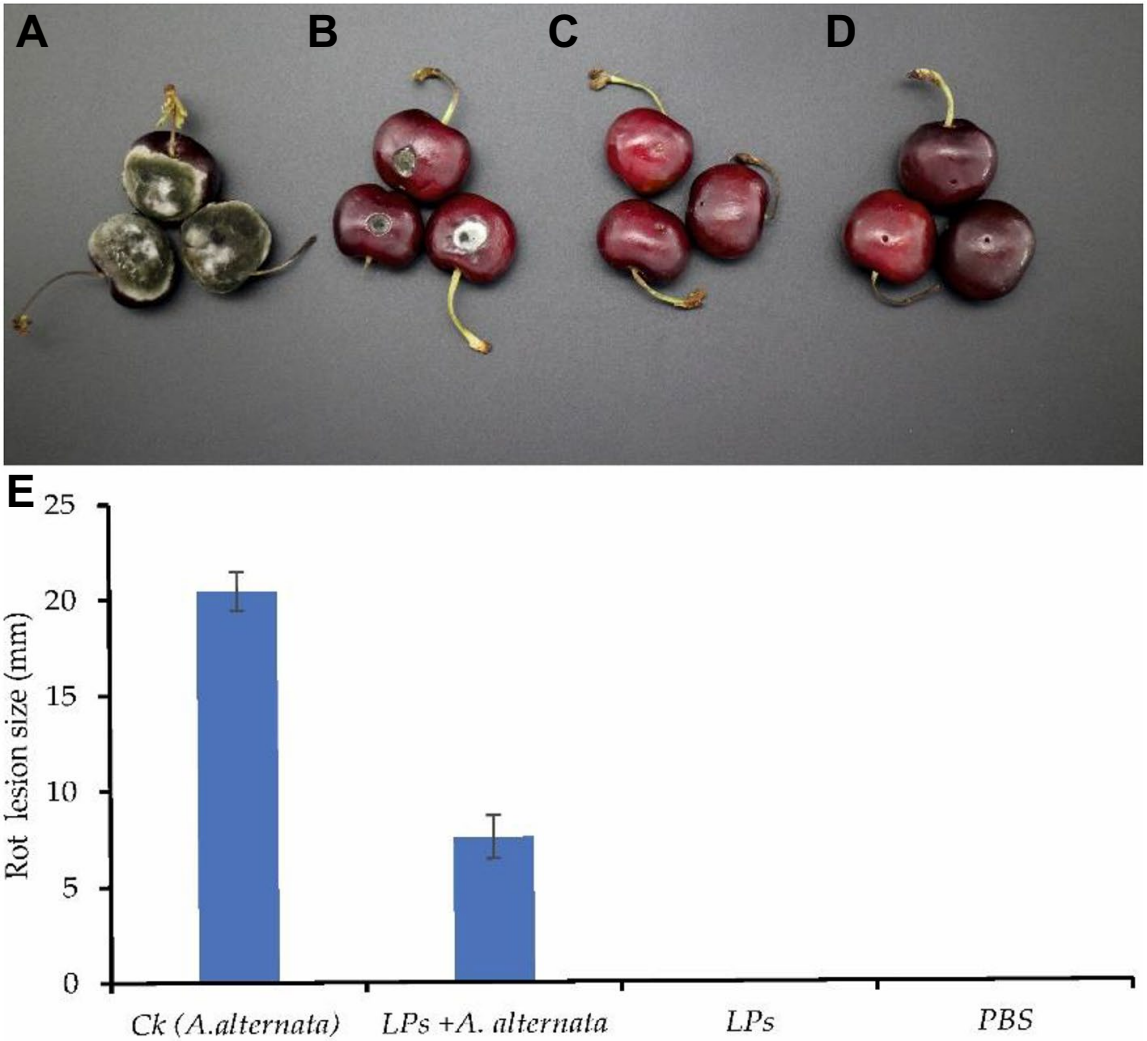
LPs from Y17B suppressed fruit rot development in cherry fruit. **(A)** Ck: cherry fruit infected with *A. alternata* only. **(B)** Treatment: cherry fruit infected with *A. alternata* and treated with LPs. **(C)** Cherry fruit treated with LPs only. **(D)** Cherry fruit treated with PBS only. **(E)** Mean values of rot lesion diameter ± SD.

*Alternaria alternata* caused typical fruit rot symptoms in all inoculated cherry fruit, with the formation of dark olive-green to whitish aerial mycelia around the wounds. Conidia production was also observed at the site of infection. The rot lesions expanded toward the surrounding area and had an average diameter of 20.4 ± 1.0 mm in untreated fruit. The application of LPs reduced the lesion size induced by *A. alternata* compared with non-treated fruit. A significant effect of 10 μl of LPs with a concentration of 300 μg/ml was observed in this treatment and lesion diameter was reduced to 7.5 ± 1.1 mm (more than 63%), as shown in Figure 10.

## 4. Discussion

Cherry is the most important fruit crop in the world, and postharvest rot of cherry fruit caused by the fungus *A. alternata* is one of the most devastating postharvest diseases, reducing the quality and quantity of fruit (Ahmad et al., 2020). Synthetic chemicals are commonly used to control fungal pathogens but excessive and regular use of these chemical agents causes environmental contamination due to their long-term residual effects. LPs produced by the *Bacillus* genus possess a strong antifungal action, which reduces the risk of postharvest fungal rots. The added advantage of LPs is that they are safe as they do not pose any health and environmental hazard (Ongena and Jacques, 2008; Ali et al., 2016).

In this study, the antifungal activity of *B. subtilis* Y17B and its LPs against *A. alternata* has been elucidated. Previous research reports state that the major reason for the antifungal activity of *Bacillus* species is the production of LPs, i.e., surfactin, iturin, and fengycin, which are synthesized by NRPSs (Romero et al., 2007; Pramudito et al., 2018). In our study, the PCR amplification of the biosynthetic genes surfactin, iturin, and fengycin from the genome of *B. subtilis* Y17B revealed the presence of gene clusters for non-ribosomal lipopeptide synthetases for surfactin, iturin, and fengycin. Several recent studies have reported that the genomes of *B.subtilus* and *B. amyloliquefaciens contain* biosynthetic genes that *encode for antifungal LPs, including* fengycin, surfactin, bacillibactin, iturin, subtilosin, and bacilysin (Farzand et al., 2019; Su et al., 2020). The presence of antifungal LPs in the crude extract of *B. subtilis* Y17B was further confirmed by UPLC Q TOF-MS analysis. The LPs were detected based on their molecular weight (m/z). Previous studies have reported the potential of *Bacillus* species for producing several antifungal LPs in response to different fungal infections, which were detected by mass spectrometry (MS) based on their molecular weight (Frikha-Gargouri et al., 2017; Farzand et al., 2019). In another instance, several antifungal LPs, including surfactin, bacillomycin, and fengycin, were detected with molecular masses of 994.64, 1031.54, and 1491.84, respectively, in *Bacillus* XT1 culture through Q TOF-MS analysis (Toral et al., 2018).

LPs produced by *B. subtilis* Y17B showed an antagonistic effect against *A. alternata* in both dual culture and on PDA amended with LPs. A recent study reported that LPs from *B. altitudinis* Q7 and *B. subtilis* BS-01 show stable antifungal activity against *A. alternata* and *A. solani*, respectively, in extreme environments and are good biological antagonists (Awan et al., 2022; Guo et al., 2022). Additionally, several studies have previously reported the antagonistic effect of LPs and VOCs of *Bacillus* species on different fungal pathogens, especially *A. alternata* (Chowdhury et al., 2015; Kaur et al., 2015; Wang et al., 2022a; Jia et al., 2023). *Bacillus* strains *B. subtilis 6,051, B. amyloliquefaciens* FZB42, and *Bacillus* XT1 CECT have been reported as biocontrol agents due to their production of antifungal LPs, which inhibit different fungi, such as *Aspergillus* species*, Fusarium graminearum*, *and Botrytis cinerea* (Bais et al., 2004; Toral et al., 2018; Hanif et al., 2019). Similar results were reported by Ali et al. (2016) who showed that the culture filtrates of *B. subtilis* and *B. amyloliquefaciens* exert strong antagonistic activity against *Alternaria* species due to the presence of the antimicrobial cyclic LP families fengycin-A and fengycin-B. In another study, LCMS analysis of the crude mixture of *B. subtilis* revealed the presence of antifungal LPs. These LPs inhibited the growth of *Verticillium dahliae* by causing cell lysis, hyphal swelling, and the downregulation of melanin-related genes. LPs inhibited fungal gene expression associated with the signaling pathway of protein catabolism and secondary metabolism (Yu et al., 2019). Another study reported a similar result in that the enhanced transcription of genes involved in flavonoid biosynthesis and accumulation of flavonoids was induced by *B. subtilis* Y2 to fight against the pathogen *A. brassicicola*, which causes black spot disease of pear fruit (Wang et al., 2023).

In this study, ultrastructural changes were observed in *A. alternata*. The LPs produced by YB17 inhibited the germination of conidia and caused alterations in the hyphal structures of *A. alternata*. SEM analysis revealed damage to the fungal structures of *A. alternata*, which appeared irregular in shape, with loose and ruptured cell walls, surface subsidence, and shriveled trunks. In agreement with our results, a previous study revealed that LPs produced by *B. subtilis* YM 10–20 cause the inhibition of conidia germination in *Penicillium roqueforti* (Chitarra et al., 2003), and LPs of *B. subtilis* BS-99-H cause alterations in the morphology of *Pestalotiopsis eugeniae*, which is associated with wax apple fruit rot (Lin et al., 2010). Several studies have shown that LPs from *Bacillus* species alter and severely damage fungal hyphae and spores (Souto et al., 2004; Etchegaray et al., 2008). Additionally, in other studies, the LPs produced by *B. amyloliquefaciens* were reported to be strong antifungal agents against *F. graminearum and F. solani* (Torres et al., 2017; Hanif et al., 2019). TEM studies showed that the cell walls of the treated hyphae were thinner than the hyphae in the untreated controls, with uneven thickness of conidia and plasmolysis and fragmentation of the plasma membranes, which represents cytoplasm leakage. Similarly, TEM images of *B. cinerea* after treatment with LPs of XT1 Lps showed damage and alteration to the ultrastructure of the fungal hyphae and ultimately cytoplasmic leakage (Toral et al., 2018). A similar study, using SEM and TEM analysis, showed that fengycin BS155 extracted from marine *B. subtilis* causes morphological changes in the plasma membrane and cell wall of *Magnaporthe grisea*. Biochemical and proteomic assays revealed that fengycin BS155 possesses the ability to reduce mitochondrial membrane potential (MMP), induce oxidative stress, and downregulate the expression level of oxidative stress-scavenging enzymes. Additionally, fengycin BS155 causes chromatin condensation in fungal hyphal cells, resulting in cell death (Zhang and Sun, 2018).

An antagonism assay in cherry fruit was also conducted to evaluate the antifungal activity of the LPs produced by *B. subtilis* Y17B against postharvest fruit rot of cherry caused by *A. alternata*. The results showed that rot disease in cherry fruit was significantly reduced by the application of LPs due to their antifungal action against *A. alternata*. A previous study suggested that the LPs from *B. subtilis* ABS-S14 act as defense-related genes to inhibit pathogen growth (Waewthongrak et al., 2014). Another study produced similar results showing that LPs produced by *B. mojavensis* P1709 are an effective tool for controlling postharvest decay and mycotoxin contamination in cherry tomatoes (Galitskaya et al., 2022). In conclusion, we report that LPs produced by *B. subtilis* strain Y17B potentially exert strong antifungal activity against *A. alternata*, causing ultrastructural damage to the pathogen and reducing the disease intensity of *A. alternata* on cherry fruit. *B. subtilis* Y17B can be formulated as a biopesticide against the postharvest fruit rot pathogen *A. alternata*. For future studies, the antifungal potential of *B. subtilis* Y17B against *A. alternata* and the degradation of the *Alternaria* mycotoxins tenuazonic acid and alternariol could be studied under the influence of purified surfactin, iturin, and fengycin. However, the exact mechanism by which purified LPs of *B. subtilis* Y17B cause fungal cell death still needs to be investigated.

## Data availability statement

The datasets presented in this study can be found in online repositories. The names of the repository/repositories and accession number(s) can be found in the article/supplementary material.

## Author contributions

TA, FX, and YL contributed to the conception and designed the experiments. TA performed the isolation, antifungal, antagonism assays, SEM and TEM analysis, and wrote the first draft of manuscript. FX, TA, and CN contributed to the identification of the strain and phylogenetic analysis. TA and YL contributed to the identification of biosynthetic genes and UPLC Q TOF-MS analysis. CC and XY analyzed the data. AM, CC, CN, XY, and YX contributed to the writing and editing of the manuscript. All authors contributed to the article and approved the submitted version.

## Funding

This research was funded by National Key Research and Development Program of China (No. 2022YFE0139500) and National Key Research and Development Program of China (No. 2019YFC1604502).

## Conflict of interest

The authors declare that the research was conducted in the absence of any commercial or financial relationships that could be construed as a potential conflict of interest.

## Publisher’s note

All claims expressed in this article are solely those of the authors and do not necessarily represent those of their affiliated organizations, or those of the publisher, the editors and the reviewers. Any product that may be evaluated in this article, or claim that may be made by its manufacturer, is not guaranteed or endorsed by the publisher.
